# Supplementary figures and images for: Ancestral Origin and Functional Expression of a Hyaluronic Acid Pathway Complement in Mussels
Source: Biology (Basel). 2025 Jul 24;14(8):930. doi: 10.3390/biology14080930 (PMC12383584; doi:10.3390/biology14080930)

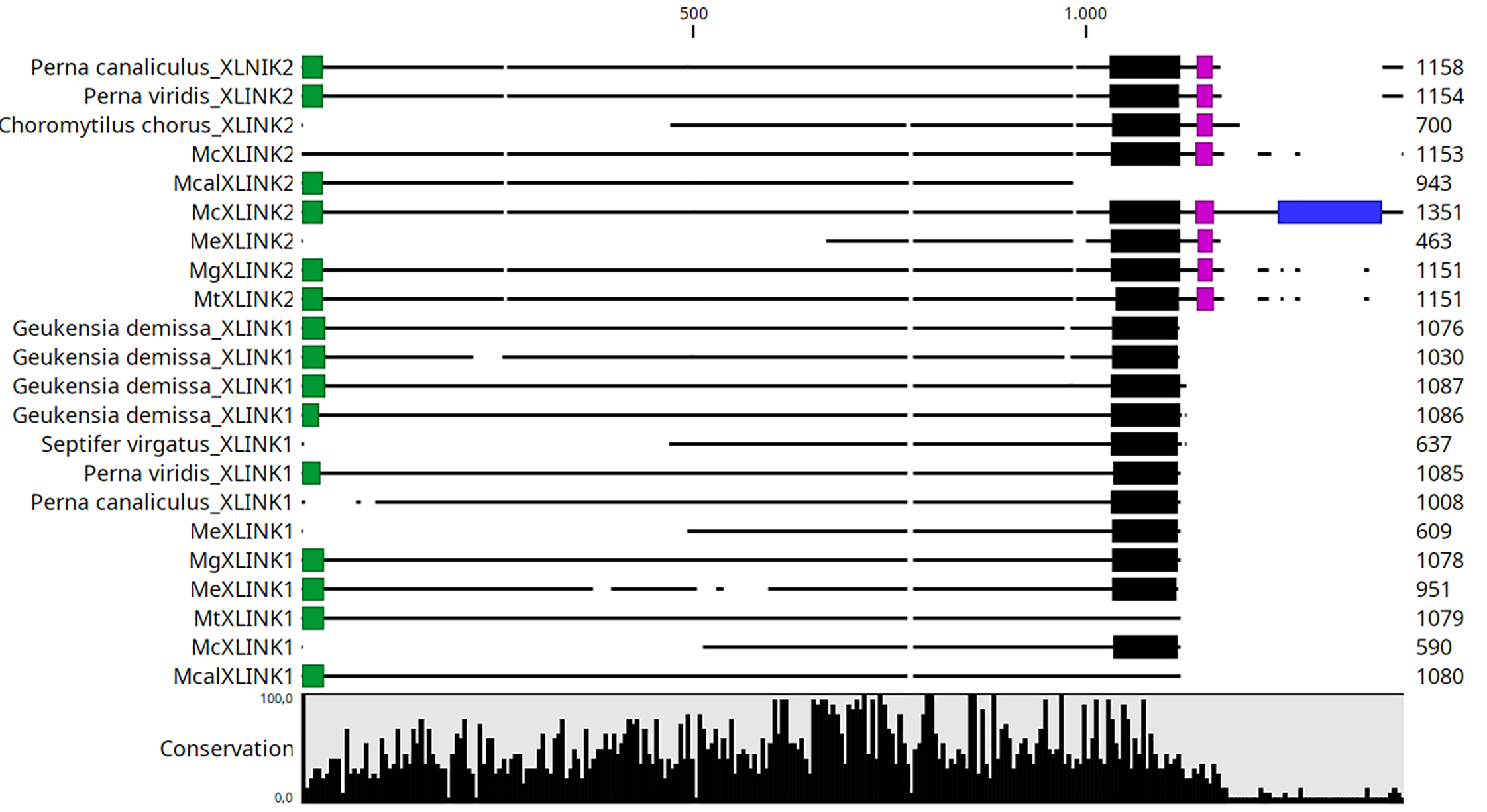

Supplement: Supplementary file 1 [file biology-14-00930-s001.zip › Figure S1.tif]

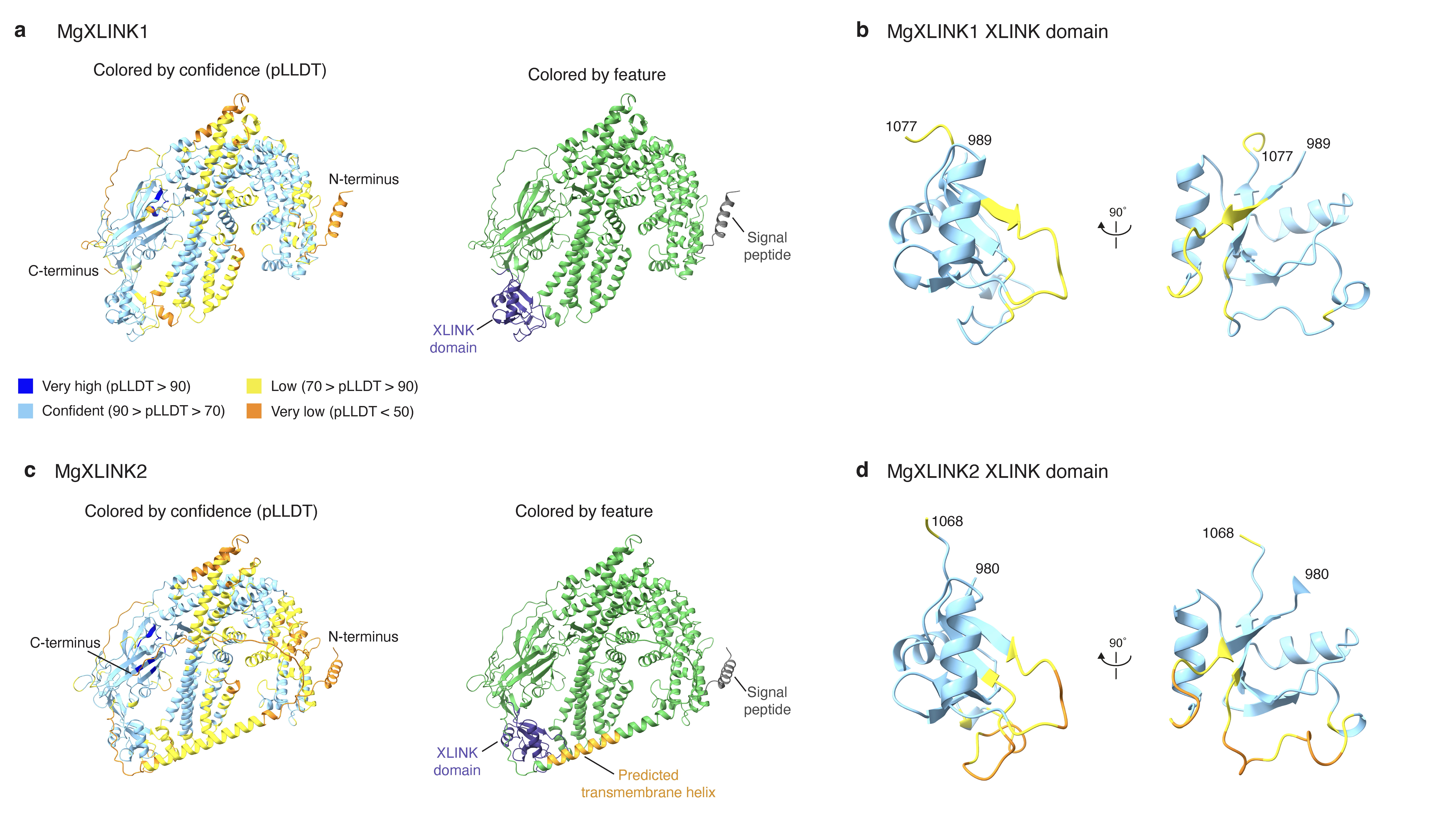

Supplement: Supplementary file 1 [file biology-14-00930-s001.zip › Figure S2.tif]

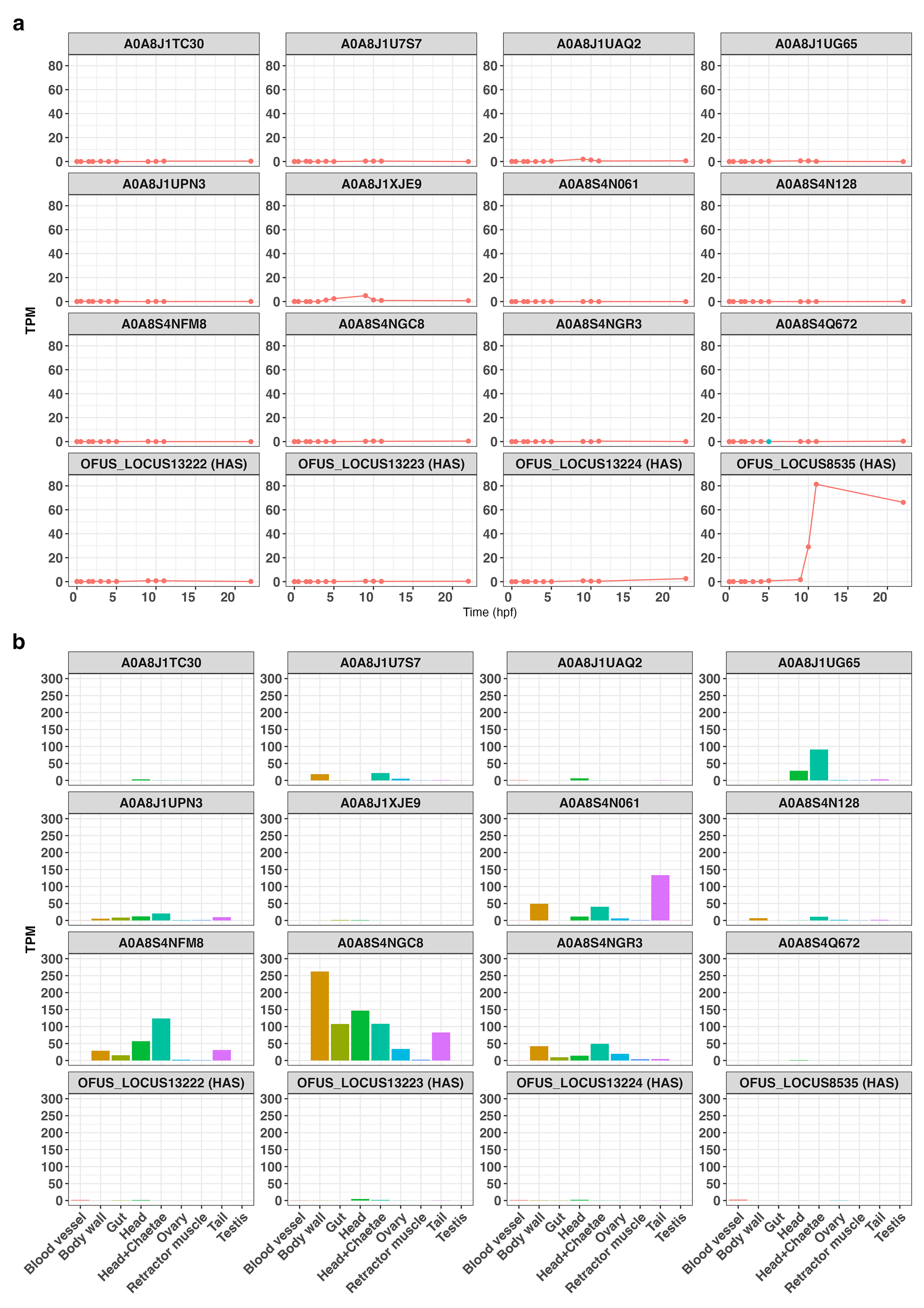

Supplement: Supplementary file 1 [file biology-14-00930-s001.zip › Figure S3.tif]
